# The Impact of Shared Governance Model's Implementation on Professional Governance Perceptions of Nurses in Saudi Arabia: A Randomised Controlled Trial

**DOI:** 10.1155/2024/7263656

**Published:** 2024-02-07

**Authors:** Mahmoud Hamdan, Amar Hisham Jaafar

**Affiliations:** ^1^King Saud Medical City, Riyadh, Saudi Arabia; ^2^College of Graduate Studies (COGS), Universiti Tenaga Nasional (UNITEN), Putrajaya Campus, Jalan IKRAM-UNITEN, Kajang 4300, Selangor, Malaysia; ^3^Institute of Energy Policy and Research (IEPRe), Universiti Tenaga Nasional (UNITEN), Putrajaya Campus, Jalan IKRAM-UNITEN, Kajang 4300, Selangor, Malaysia; ^4^College of Business Management and Accounting (COBA), Universiti Tenaga Nasional (UNITEN), Putrajaya Campus, Jalan IKRAM-UNITEN, Kajang 43000, Selangor, Malaysia

## Abstract

**Objective:**

This study aimed to evaluate the impact of the shared governance model application on the level of perceived professional governance among clinical nurses in a tertiary hospital in Riyadh.

**Background:**

Professional governance continues traditional governance, shared governance, and self-governance. Shared governance (SG) is the engagement of clinical nurses in decision-making at different levels. This empowers nurses, increases job satisfaction, improves clinical outcomes, and enhances patient satisfaction.

**Methods:**

This randomised control trial in which researchers distributed the Index of Professional Nursing Governance (IPNG) to a random sample of 440 nurses working in a 1200-bed tertiary hospital in Riyadh and divided into experimental and control groups. The intervention included designing and implementing a nursing shared governance model at the hospital level; professional governance was measured before and eight months after implementation. The IPNG was used to measure nurses' perceived level of professional governance before and after the intervention. The sample was divided into experimental and control groups.

**Results:**

By comparing experimental and control groups, there was no statistically significant difference between them regarding professional governance subscales and the total IPNG scores before the intervention. At the same time, there was a considerable difference between them after the intervention. Moreover, the scores of the six professional governance subscales and the overall IPNG scores significantly increased after the intervention in the experimental group. They showed no significant difference in the control group.

**Conclusion:**

Designing and implementing specific shared governance structures and processes effectively enhanced nurses' perceived level of shared governance at the hospital, as evidenced by significantly higher postintervention IPNG scores. Elements of the shared governance model that proved effective included engaging nurses in decision-making at various organizational levels and empowering their involvement.

## 1. Introduction

### 1.1. Background

Shared governance is a professional practice model that decentralizes decision-making in healthcare organizations by engaging frontline clinical nurses in a structural framework for participating in organizational oversight, policy setting, and other administrative functions that directly impact their practice and work environment. The key principles of shared governance include empowering nurses through a collaborative management structure that gives them authority and control over practice-related decisions while also fostering a greater sense of responsibility and accountability. At its core, shared governance aims to actively engage staff nurses beyond their traditional clinical roles by providing opportunities to have a meaningful voice and influence in the organizational decisions that govern their profession [1, 2].

Moreover, professional governance is a broader term involving the continuum of traditional, shared, and self-governance. Control over practice and resources moves gradually from management to clinical nurses in this continuum. The Index of Professional Nursing Governance (IPNG) is a valid and reliable tool used to measure it [3]. Previous research has shown the advantages of shared governance in nursing in the past few decades; it has been applied in various ways to hospital nurses. Improved nurse retention, work satisfaction, nursing-sensitive indicators, and patient satisfaction were some of these advantages [4–9].

Building a culture of shared governance supports nurses to be effective in decision-making, reducing centralisation in decision-making, increasing confidence and accountability, and creating a collaborative relationship between nurses and other healthcare professionals [10]. This is expected to enhance outcomes for nurses and patients [3, 11]. The shared governance framework is usually applied by moving from a hierarchical to a councillor model, which enhances the involvement of clinical nurses in decision-making [12].

### 1.2. Theoretical Framework

Kanter's theory of structural empowerment is considered a theoretical basis for nursing governance. According to the idea, structurally empowered nurses can achieve more due to their access to information, resources, support, and development opportunities [13, 14]. The theoretical underpinning of structural empowerment through shared governance models is directly linked to improved nursing and patient outcomes. This relationship between empowerment structures and positive results forms the basis of the Magnet recognition program developed by ANCC for hospitals in the US. While Saudi Arabia does not have an equivalent national accreditation system akin to Magnet, implementing shared governance principles aligned with Kanter's empowerment theory could still yield benefits seen in other contexts. The aim of this study is to explore if introducing shared governance councils in a Saudi hospital impacts nurses' perceptions of professional autonomy and influence, as measured by the IPNG tool, even without the external incentive of Magnet designation. Clarifying this connection and focus on potential internal outcomes rather than accreditation would better contextualize the rationale for the study within the Saudi healthcare system [7, 8].

Furthermore, structural empowerment is one of the main components of the Magnet® Model, and this Model is considered the heart of the American Nurses Credentialing Center (ANCC) Magnet® Recognition Program [15]. Magnet® hospitals or hospitals preparing for Magnet® apply this Model and usually implement shared governance frameworks to enhance nurses' involvement and improve outcomes related to nurses and patients [8, 9, 11]. There are three Magnet hospitals in Saudi Arabia, but several are still on their journey toward Magnet® [16]. Studies investigating the effect of implementing the shared governance model on nurses' professional governance level are limited. This research has addressed how nurses' engagement in shared governance structures and processes significantly increase their professional governance. Kanter's theory of structural empowerment serves as the theoretical basis, proposing that empowering organizational structures like shared governance lead to greater job satisfaction and effectiveness. By decentralizing decision-making and giving nurses governance council roles, shared governance aims to structurally empower frontline staff. This study hypothesizes that implementing such a model will increase nurses' perceived professional governance as measured by the IPNG tool, in line with Kanter's empowerment concepts.

### 1.3. Research Question

1. What is the impact of the shared governance model application on the level of perceived professional governance among clinical nurses in a tertiary hospital in Riyadh?

## 2. Materials and Methods

### 2.1. Study Design

This study employed a randomized control trial design to evaluate the impact of implementing a shared governance model on nurses' perception of professional governance.

### 2.2. Study Groups

This randomised control trial measured the level of professional governance among a control group and an experimental group of clinical nurses before applying shared governance structures and processes in the hospital and 8 months after the implementation. Nurse participants were engaged in the shared governance model through participation in the main shared governance councils or unit-based councils (UBCs).

### 2.3. Inclusion Criteria

The inclusion criteria selected clinical nurses working at the tertiary hospital in Riyadh, Saudi Arabia. The nurses needed to have a minimum of three months of clinical nursing experience in order to have adequate exposure to the clinical environment. All participating nurses were also required to be permanently licensed. Another inclusion requirement was that nurses provided informed consent after being made aware of the study's aims and objectives. Only those nurses who completed both the preintervention and postintervention questionnaires, which assessed perceptions of professional governance before and after the implementation of the shared governance model, respectively, were fully included. By limiting inclusion to clinically active and experienced nurses who consented and could have their perceptions measured both longitudinally, the criteria aimed to comprise a sample population most appropriately suited for the study.

### 2.4. Exclusion Criteria

Nurses were excluded if they had less than 3 months of clinical experience, as they were still new to the clinical setting. Additionally, nurses who did not provide direct patient care, such as those in solely administrative roles, were excluded. Nurses who were on extended leave during the 8-month study period or who could not understand or speak English sufficiently to complete the English questionnaire were also excluded.

### 2.5. Study Participants

For this randomised control research, 440 clinical nurses from a variety of specialities were selected using a systematic random sampling process. The estimated sample size was determined using the Power Primer with a medium effect size, power of 0.80, and of 0.05 [14]. Using Microsoft Excel, randomisation was carried out by choosing every tenth nurse from a list of 2206 nurses. A permanent licensed nurse with a clinical employment in any hospital and at least three months of experience was the basis for recruiting the participants. Before receiving consent, the participants were informed of the study's aims and objectives. Before and after the intervention, the recruited nurses willingly consented to express their opinions. The initial sample size selected was 440 clinical nurses through systematic random sampling. However, attrition occurred over the course of the study. In the experimental group, 16 nurses withdrew participation and an additional 4 only partially completed the postintervention questionnaire, reducing that group's final sample to 200 nurses. In the control group, 19 nurses withdrew from the study, resulting in a final sample of 200 nurses who completed questionnaires at both time points. In total, the final sample size that completed the study was 200 nurses in each control and experimental groups, for a total of 400 nurses.

### 2.6. Study Setting

**Figure 1:**
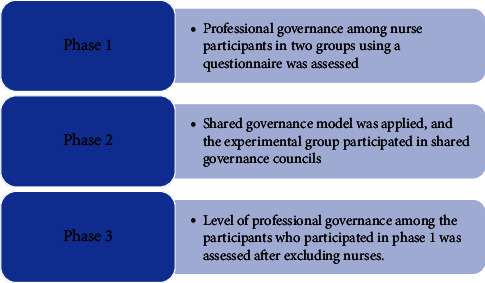
Phases of model implementation (source: author).

The study took place in a tertiary hospital in Riyadh, Saudi Arabia. A random sample of 440 clinical nurses from different specialties was recruited for this study and divided equally into two control and experimental groups. The research was conducted over eight months in three phases (see Figure 1). In phase 1, the researchers assessed the baseline level of professional governance among the nurse participants in the two groups using a questionnaire tool. In phase 2, the shared governance model, including structures and processes, was applied, and the experimental group nurses were supported to participate in the shared governance councils. In phase 3, the researchers reassessed the level of professional governance among the participants who participated in phase 1. 16 nurses withdrew from the experimental group, and four participants were also excluded from the experimental group because they only partially answered the study questionnaire. In addition, 19 nurses were excluded from the control group because they withdrew in phase 3.

#### 2.6.1. Model Implementation

**Figure 2:**
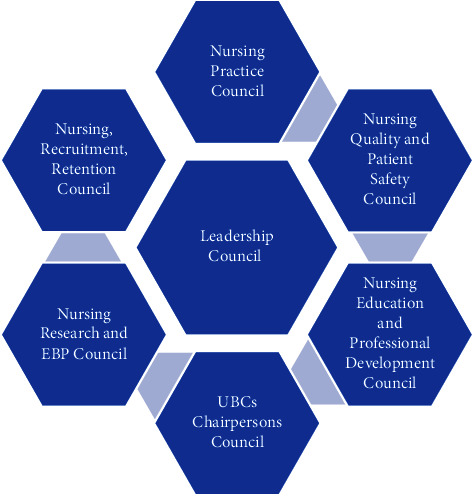
Shared governance model (source: author).

The design of the nursing shared governance model (see Figure 2) was derived from references related to the American Nurses Credentialing Center (ANCC) as well as the published shared governance experiences by Magnet hospitals and experts [12, 17–21]. The Model included seven main shared governance councils; the practice council, quality and patient safety council, education and professional development council, recruitment and retention council, research and evidence-based practice council, leadership council, and unit-based council (UBC) chairperson's council. The study selected a shared governance model from Magnet hospital literature and aligned with Kanter's empowerment theory, matching the theoretical framework. While not a Magnet facility, the hospital structure mirrored those achieving positive shared governance outcomes through councils empowering frontline involvement in decisions. Prior to implementation, staff reviewed the model for local relevance, customizing as needed. Its representation of staff and collaborative decision-making directly addressed assessing governance perceptions. Together, these links adequately justified applying this conceptual model to evaluate the intervention's influence.

In addition, a unit-based council (UBC) was developed in each nursing unit. The number of nurse members in each main shared governance council or UBC ranges from 5 to 12 members based on the capacity of the nursing unit. Furthermore, commonly shared governance bylaws were developed to regulate the hospital's structures, processes, and pathways of shared governance. This included the path and support of the ideas for improvement (IFIs) submitted by the UBCs. A charter was developed for each council to clarify the scope and main functions of the committee. The leadership council coordinated the efforts of the main shared governance councils and was led by the chief nursing officer. In addition, the main shared governance councils were led by senior nurse managers. However, the UBC chairpersons, members, and members of the main shared governance councils were clinical nurses.

Nurse managers and clinical nurses attended extensive awareness sessions about shared governance, including the new shared governance model, structures and processes, and the benefits of participating in shared governance councils for clinical nurses and the hospital. These awareness sessions helped introduce this major change to the clinical nurses. They motivated many of them to join the committees despite the current challenges of the nursing shortage and high workload. In addition, the sessions helped improve the tendency of nurse managers to maintain control of decision-making. Head nurses and nurse managers were encouraged to attend the UBC meetings as nonvoting members. Moreover, they supported the UBC members by facilitating their meetings and providing them with their time in shared governance activities. The experimental group nurses attended the awareness sessions and were motivated to participate in the main shared governance councils and the UBCs.

#### 2.6.2. Data Collection

The questionnaire started with a demographic profile section. It was designed to encourage nurses to disclose their personal information related to this study to promote their completion of the questionnaire [22]. This section included questions for age, gender, marital status, length of service in a nursing career, working unit, size of service in the current working team, and highest educational qualification.

The Index of Professional Nursing Governance (IPNG 3.0) short form was used in this study. The tool was developed by Robert Hess (1998) and has been widely used to measure professional governance among nurses [23].

The IPNG includes six subscales and 50 items measuring nurses' governance perception. The overall score categorises governance as traditional governance when the total score ranges from 50 to 100, shared governance when the total score ranges from 101 to 200, and self-governance when the total score ranges from 201 to 250. This instrument uses a five-point Likert scale, which includes score 1 as nursing management/administration only, score 2 as primarily nursing management/administration with some staff nurse input, score 3 as equally shared by staff nurses and nursing management/administration, score 4 as especially staff nurses with some nursing management/administration and score 5 as staff nurses only. Scores 1 and 2 reflect that decision-making is dominated by management/administration. Scores of 4 and 5 indicate that nurses participate more in decision-making.

The IPNG 3.0 short version contains six subscales of professional governance. First, nursing personnel assesses who controls nursing personnel and related structures, including 12 items. Second, information assesses who accesses the information related to governance activities and includes nine items. Next is the Resources subscale, which measures who has influence over hospital resources and consists of nine things. This is followed by the subscale of participation, which assesses who participates in shared governance structures at the unit and hospital levels and includes eight items. Next is practice, which measures who has control over the professional course and consists of seven things. Finally, the dimension of Goals contains five items, which assess who sets goals and negotiates conflict solutions. Data collection used the English version of the tool since the English language is the official language in the hospital and is used in communications, meetings, documentation, and handoff. The test-retest reliability test showed good reliability (*r* = 0.857), and internal consistency reliability was excellent (*a* = 0.966). The applied longitudinal design may affect the recall bias of the nurse participants, but this was minimised by shortening the period between study phases.

#### 2.6.3. Data Analysis

Data analysis was performed using the SPSS® software version 25 through the descriptive statistical measures of frequency distribution, mean, and standard deviation. Normality was assessed via the Kolmogorov–Smirnov test, which was insignificant (*p* > 0.05). Therefore, the parametric tests were applied for data analysis. To verify the study intervention, independent samples *t*-test and paired sample *t-*test were performed to compare the scores of professional governance subscales before and after the intervention. The significance level was set at less than 0.05.

### 2.7. Ethical Considerations

The Institutional Review Board of King Saud Medical City authorised the research. The IRB Registration Number with KACST, KSA is H-01-R-053. The hospital administrators and nursing department heads within the same health organisations also endorsed the visits. Each participant received a cover letter detailing the instruments' purpose, significance, content, instructions, and completion time. After reading the cover letter, each individual who agreed to participate in the study completed the informed consent form. Participants were free to quit the study at any time without penalties since participation was entirely voluntary.

To protect confidentiality, identifying information was removed from surveys before data entry and each participant was assigned a unique study ID number. Electronic data files and physical consent forms/surveys were securely stored in locked cabinets with limited access for research staff only. Final deidentified datasets did not contain any direct identifiers to allow for anonymous analysis and reporting of aggregate findings.

## 3. Results

### 3.1. Reliability

The IPNG Total Score and Factor Subscales were assessed for internal consistency using the test-retest reliability test.  Table 1 demonstrated excellent reliability and the internal consistency of all the components, including personnel (*r* = 0.962), information (*r* = 0.957), participation (*r* = 0.953), and goals (*r* = 0.949), as Cronbach alpha was more than 0.9. Besides, because the Cronbach alpha value ranged from 0.8 to 0.89, the internal consistency and dependability of resources (*r* = 0.860) and practice (*r* = 0.872) were both good.

### 3.2. Comparison of Demographic Variables between the Two Groups

Most nurse participants were females, in the age group of 31 to 40 years, had a bachelor's degree, had an overall experience of between 6 to 10 years, and had experience in the current working unit between 1 and 5 years. In terms of the operational teams, they were equally divided between critical care units and noncritical care units. By comparing the demographic variables between the experimental and control groups, results indicate there was no significant difference between them (*p* > 0.05) (Table 2).

In Table 2, shared governance ranges are specified for each professional governance subscale and the overall professional governance score. For the level of professional governance measured for the nurse participants before the intervention for both groups, the mean professional governance scores were almost on the lower shared governance limits for each of the subscales except for personnel and participation subscales which were lower than the shared governance zone (traditional governance). Independent samples *t*-test was used to examine the difference between the two groups before and after intervention regarding professional governance subscales and the overall IPNG scores. Results show that difference between the two groups in terms of professional governance subscales is not significant and the total IPNG scores before the intervention (*p* > 0.05). However, the mean scores after intervention for the experimental group are significantly higher than those for the control group for all professional governance subscales and the total IPNG scores (*p* < 0.001).

### 3.3. Impact of the Shared Governance Model on Perceived Professional Governance among Clinical Nurses before and after Intervention

Both independent and paired sample *t*-test was used to compare the scores of the professional governance subscales and the total IPNG scores before and after the intervention. Mean scores of professional governance subscales and the full IPNG scores show no statistical significant difference before and after the intervention for the control group (*p* > 0.05), while the scores increased significantly for the experimental group after the intervention for the subscales as well as the total IPNG scores (*p* < 0.001) (Tables 3 and 4).

## 4. Discussion

The hospital of this study has several multidisciplinary committees that make decisions on issues related to their scope. Members of these committees are usually leaders and managers, with minimal involvement of clinical nurses. However, the new shared governance structures and processes considerably involved clinical nurses enormously and supported them to be involved in making decisions related to their practice. These nurses were supported to receive, review, and discuss data related to their unit, including nursing-sensitive indicators, patient satisfaction, and nurses' job satisfaction.

The findings of this study showed that the baseline professional governance scores among clinical nurses were at the lower shared governance limits for the majority of the professional governance subscales as well as the overall scores. This is consistent with the findings of studies conducted in Saudi Arabia, Jordan, Lebanon, Egypt, and the United States [8, 9, 11, 24–26].

The mean scores of professional governance subscales after the intervention for the experimental group are significantly higher than those for the control group. In addition, the mean scores of professional governance subscales after the intervention are considerably higher than the mean scores before the intervention for the experimental group (*P* < 0.001). This indicates that the professional governance perceptions of clinical nurses improved after implementing the shared governance model and engaging nurses in the shared governance councils and processes. These findings confirm to a longitudinal study conducted in the US, in which the researchers measured professional governance among nurses in 2013, 2015, and 2017 using the IPNG [26].

Since the hospital had no proper shared governance structures in the first two surveys, results showed total governance scores of 168.62 and 168.39 using IPNG 2.0, below the shared governance zone. After applying better-shared governance structures, the entire shared governance score was 103.84 using IPNG 3.0 within the shared governance zone. Therefore, measuring shared governance among nurses, especially when changes occur, is recommended. Implementing a shared governance model improves nurses' perceptions of professional authority and enhances the culture of shared governance.

This study's findings also confirm the results of a quasiexperimental study conducted in the US, in which the scores of shared governance dimensions increased significantly after enhancing shared governance structures and processes [19]. The interventions included (1) interprofessional strategic planning retreats every six to eight months, (2) celebrating shared governance achievements annually, (3) developing three new unit-based councils as part of the hospital's shared governance structure, (4) increasing nurses' access to the hospital library as well as shared governance paid time, (5) enhancing nurses' involvement in budgeting, and (6) submitting annual goals and quarterly progress reports to the coordinating council, and training the councils so they can conduct effective, shared governance meetings. Moreover, according to a longitudinal study in which professional governance perceptions among clinical nurses were measured in 2012 and 2015, professional governance increased after enhancing the shared governance model and allowing nurses to participate in decision-making on different levels [27].

Once the shared governance model was designed and approved, it was announced to all nurses. In addition, nurse managers and clinical nurses attended extensive awareness sessions about the Model and the pathways. Moreover, the availability of unit-level data was helpful for the UBCs to ensure that their IFIs were data-driven. Furthermore, head nurses and nurse managers facilitated the UBC meetings, attended the meetings as nonvoting members, and supported clinical nurses by giving them the time they spent in shared governance activities back to them. This helped support shared governance and was consistent with the findings of the study conducted in Finland to describe factors that support or obstruct shared administration. Semistructured interviews revealed nurse managers' support, enthusiastic personnel, and neighbouring universities supported shared governance. However, blocking factors included lack of time, poor understanding, and insufficient skills [28]. When clinical nurses are engaged in shared governance, and their ideas of improvement are supported, improvement is expected in nursing-sensitive indicators, patients' satisfaction, and nurses' job satisfaction [6–8].

## 5. Strengths and Limitations

One of the key strengths of the study is that it provides practical implications and solutions for designing and implementing shared governance structures and processes to enhance shared governance among clinical nurses. Furthermore, the main shared governance councils and the unit managers supported their ideas for improvement. Since the hospital was in the early stages of its journey toward Magnet®, hospital leaders were committed to applying a shared governance model to reap its benefits for patients and nurses. The sample was predominately female, reflecting the typical gender bias of this research and accurately representing the target demographic. Using a self-reported questionnaire raised the possibility of prejudice and impaired the impartiality of the nurses' replies. Yet, the researchers used an experimental design, random sampling, and a valid and reliable tool to assess clinical nurses' perspectives of professional governance. In addition, the research validated the use of a focused intervention to improve shared governance and presented a replicable model.

## 6. Conclusion

This study evaluated the effectiveness of the shared governance model's implementation among clinical nurses on their perceived level of professional governance. The Model's performance effectively improved the shared governance level among clinical nurses. These findings are of great value for hospitals struggling with lacking or ineffective shared governance structures. Applying the shared governance model successfully improved the six dimensions of professional governance among the nurse participants. A more extensive expansion to this study would be to measure the impact of shared governance on nursing-sensitive indicators, patient satisfaction, and nurses' job satisfaction.

## 7. Implications

The results of this study indicate that implementing a shared governance model can significantly empower nurses and increase their perceptions of professional autonomy and involvement in decision-making. By establishing formal councils and unit-based committees that give clinical nurses a strong voice, shared governance appears to foster increased collaboration between frontline staff and managers. When nurses feel respected and that their perspectives directly impact important areas like patient care, staffing, and quality improvement, it can boost morale and retention. This is highly relevant for other healthcare organizations aiming to combat issues like heavy workloads and shortages that undermine the nursing workforce. In the future, longitudinal measurement of governance perceptions may show that significant change can be achieved through carefully designed shared governance frameworks that make use of the tools and procedures this study has shown to be successful. Overall, restructuring toward shared models of nursing leadership holds promise for advancing practices that better engage and support bedside clinicians.

## Figures and Tables

**Table 1:** Internal consistency of the IPNG total score and factor subscales.

	Items	Cronbach's alpha (*r*)
Total instrument	50	0.979
Factor subscales		
Personnel	12	0.962
Information	9	0.957
Resources	9	0.860
Participation	8	0.953
Practice	7	0.872
Goals	5	0.949

**Table 2:** Comparison of demographic variables between the two groups.

Variable	Scale	Experimental group	Control group	*p* value (>0.05)
Age (year)	21–30	51 (25.5)	50 (24.9)	>0.05
	31–40	86 (43)	84 (41.8)	>0.05
	41–50	46 (23)	49 (24.3)	>0.05
	51–60	17 (8.5)	18 (9)	>0.05

Gender	Male	2 (1)	0 (0)	>0.05
	Female	198 (99)	201 (100)	>0.05

Education	Diploma	18 (9)	15 (7.5)	>0.05
	Bachelor	157 (78.5)	160 (79.6)	>0.05
	Postgraduate	25 (12.5)	26 (12.9)	>0.05

Overall experience (year)	1–5	19 (9.5)	18 (9)	>0.05
	6–10	87 (43.5)	85 (42.3)	>0.05
	11–15	24 (12)	26 (12.9)	>0.05
	>15	70 (35)	72 (35.8)	>0.05

Working unit	Critical care	101 (50.5)	102 (50.7)	>0.05
	Noncritical care	99 (49.5)	99 (49.3)	>0.05

Experience in the current unit (year)	1–5	76 (38)	74 (36.8)	>0.05
	6–10	61 (30.5)	59 (29.4)	>0.05
	11–15	38 (19)	39 (19.4)	>0.05
	>15	25 (12.5)	29 (14.4)	>0.05

**Table 3:** Results of shared governance subscales and overall IPNG scores before and after intervention (independent sample *t*-test).

Variable	Group	Before intervention			After intervention		
		Mean	SD	*p* value	Mean	SD	*p* value
Personnel (25–48)	Experimental	18.91	9.29	>0.05	31.93	15.40	<0.001
	Control	18.96	9.27		18.84	9.38	

Information (19–36)	Experimental	20.87	6.49	>0.05	28.96	10.52	<0.001
	Control	20.92	6.61		20.89	6.59	

Resources (19–36)	Experimental	22.46	7.14	>0.05	28.28	8.71	<0.001
	Control	22.43	7.02		22.30	7.04	

Participation (17–32)	Experimental	14.87	6.77	>0.05	23.27	9.56	<0.001
	Control	14.89	6.78		15.00	6.87	

Practice (15–28)	Experimental	16.00	5.40	>0.05	21.98	6.92	<0.001
	Control	16.12	5.40		15.99	5.40	

Goals (10–20)	Experimental	11.24	4.17	>0.05	14.47	6.43	<0.001
	Control	11.28	4.12		11.23	4.11	

Total IPNG score (101–200)	Experimental	104.36	31.31	>0.05	148.91	49.81	<0.001
	Control	104.63	31.32		104.26	31.63	

**Table 4:** Results of shared governance subscales and overall IPNG scores in the experimental and control groups before and after intervention (paired *t*-test).

Variable	Group	Before intervention		After intervention		*p* value
		Mean	SD	Mean	SD	
Personnel (25–48)	Experimental	18.91	9.29	31.93	15.40	<0.001
	Control	18.96	9.27	18.84	9.38	>0.05

Information (19–36)	Experimental	20.87	6.49	28.96	10.52	<0.001
	Control	20.92	6.61	20.89	6.59	>0.05

Resources (19–36)	Experimental	22.46	7.14	28.28	8.71	<0.001
	Control	22.43	7.02	22.30	7.04	>0.05

Participation (17–32)	Experimental	14.87	6.77	23.27	9.56	<0.001
	Control	14.89	6.78	15.00	6.87	>0.05

Practice (15–28)	Experimental	16.00	5.40	21.98	6.92	<0.001
	Control	16.12	5.40	15.99	5.40	>0.05

Goals (10–20)	Experimental	11.24	4.17	14.47	6.43	<0.001
	Control	11.28	4.12	11.23	4.11	>0.05

Total IPNG score (101–200)	Experimental	104.36	31.31	148.91	49.81	<0.001
	Control	104.63	31.32	104.26	31.63	>0.05

## Data Availability

The data used to support the findings of this study are available from the corresponding author upon request.
